# Distance to the Scaling Law: A Useful Approach for Unveiling Relationships between Crime and Urban Metrics

**DOI:** 10.1371/journal.pone.0069580

**Published:** 2013-08-05

**Authors:** Luiz G. A. Alves, Haroldo V. Ribeiro, Ervin K. Lenzi, Renio S. Mendes

**Affiliations:** Departamento de Física and National Institute of Science and Technology for Complex Systems, Universidade Estadual de Maringá, Maringá, Brazil; University of Maribor, Slovenia

## Abstract

We report on a quantitative analysis of relationships between the number of homicides, population size and ten other urban metrics. By using data from Brazilian cities, we show that well-defined average scaling laws with the population size emerge when investigating the relations between population and number of homicides as well as population and urban metrics. We also show that the fluctuations around the scaling laws are log-normally distributed, which enabled us to model these scaling laws by a stochastic-like equation driven by a multiplicative and log-normally distributed noise. Because of the scaling laws, we argue that it is better to employ logarithms in order to describe the number of homicides in function of the urban metrics via regression analysis. In addition to the regression analysis, we propose an approach to correlate crime and urban metrics via the evaluation of the distance between the actual value of the number of homicides (as well as the value of the urban metrics) and the value that is expected by the scaling law with the population size. This approach has proved to be robust and useful for unveiling relationships/behaviors that were not properly carried out by the regression analysis, such as 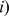 the non-explanatory potential of the elderly population when the number of homicides is much above or much below the scaling law, 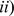 the fact that unemployment has explanatory potential only when the number of homicides is considerably larger than the expected by the power law, and 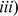 a gender difference in number of homicides, where cities with female population below the scaling law are characterized by a number of homicides above the power law.

## Introduction

The study of social complex systems has been the focus of intense research in the last decades [1]–[3]. Elections [4], [5], population growth [6], [7], economy [8]–[10], and language [11]–[13] are just a few examples of social activities that have been recently investigated. Such investigations are expected to provide a better understanding of how our society is organized and also to point out better strategies for resource management, service allocation, and political strategies. In this social context, crime is one of the most worrying activity for our society and to understand and to prevent crime acts is a huge challenge [14]–[16]. Moreover, since nowadays more than a half of the human population lives in cities [17], [18], it is crucial to analyze possible connections between criminality and urban metrics.

In fact, there exist several works that point out relationships between the number of crime acts and urban indicators such as income, unemployment and inequality [19]–[23]. Most of these papers employ regression analysis, where the dependent variable is the crime indicator (usually the number of a particular crime act) and the independent variables are urban indicators [24]–[33]. However, most of these studies does not take into account the functional form of the relationships between crime, urban indicators and the population; usually assuming these relationships to be linear [34]. On the other hand, several works have shown that crime and urban indicators obey scaling laws with the population size of the cities and also between themselves [35]–[39]. For instance, the number of homicides grows super-linearly with the population [39], [40]. Do not consider these scaling laws may be one of the reasons that several regression-based analysis led to controversial conclusions [34]. Furthermore, if we assume that these scaling laws with the population size are somehow a natural expression of how cities are organized, accounting for the scaling phenomenon is also very important for achieving a fairer comparison between cities with different population sizes.

Here we investigate a procedure that may help to solve this problem. The approach consists of defining a “distance” between the crime or urban indicators and the main tendency expected by the scaling laws with the population size. This approach is based on the recent idea of relative competitiveness proposed by Podobnik *et al.* [41] in the economic context. Our paper is thus organized as follows. We start by presenting our data of urban and crime indicators of Brazilian cities and also an intensive characterization of the scaling laws existing between these indicators and the population size. We also employ a linear regression model for explaining the number of crime acts (homicides) in terms of the urban indicators. Next, we use the previously-discussed distance in an attempt to investigate relationships/patterns between crime and urban metrics that do not appear in the regression analysis. Finally, we present a summary of our results.

## Materials and Methods

### Data presentation

We have accessed data of the Brazilian cities in the year of 2000 made freely available by the Brazil's public healthcare system – DATASUS [42]. These data are also attached to our paper in Table S1. Here, despite there being other definitions [43], we have considered that cities are the smallest administrative units with a local government and it is not our intention to discuss the role of other definitions. The data consist of the population size (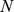) and the number of homicides (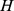) as well as ten urban indicators (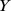) at city level: number of cases of child labour, elderly population size (older than 60 years), female population size, gross domestic product (GDP), GDP per capita, number of illiterate (older than 15 years), average family income, male population size, number of sanitation facilities, and number of unemployed (older than 16 year). More details about urban indicators can be found in Text S1. Observe that we have chosen the number of homicides as our crime indicator. This is a widely used choice [39] due the fact that homicide data are more reliable, since this ultimate expression of violence is almost always reported. Also, our ten urban indicators are usually listed as crime determinants [34]. Furthermore, we have considered only cities with at least one case of homicide in our analysis.

## Results and Discussion

### Scaling laws between crime, urban metrics and population

**Figure 1:**
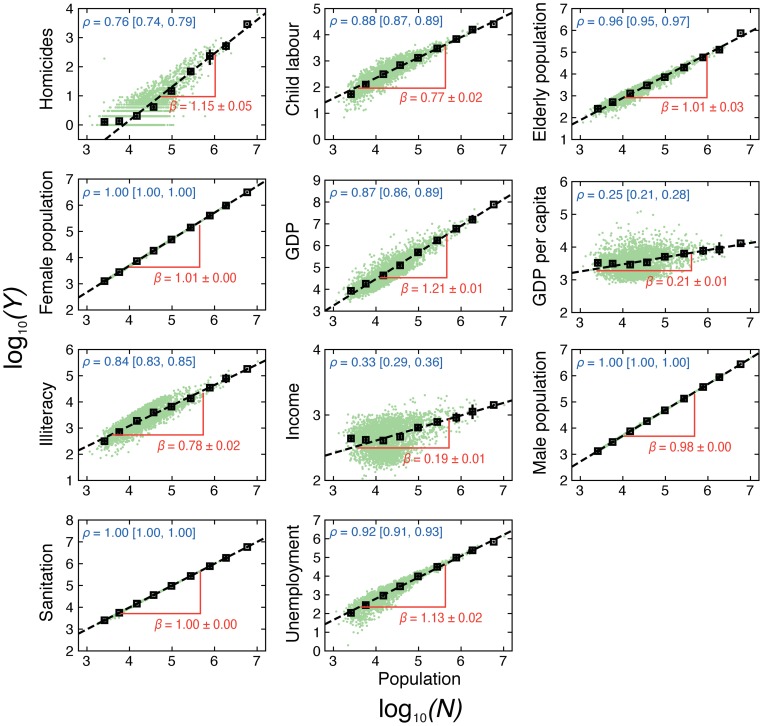
Scaling laws between the population size and the urban indicators. In each plot, the green dots are base-10 logarithmic of the values of the urban indicator (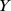) versus the population size (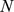) for a given city. The black squares are average values of the data binned in 10 equally spaced windows and the error bars are 95% confidence intervals for these average values obtained via bootstrapping [44]. The values of the Pearson correlation coefficients 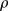 (as well as the 95% confidence intervals) of these relationships are shown in each plot. The straight dashed lines are linear fits (by least square method) to the average relationships and the slope of these lines are equal to the power law exponent 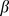 (shown in each plot).

We start by revising the question of whether homicides and urban metrics present scaling relations with the population size (see also Refs. [35]–[40]). For the sake of simplicity, let us denote the population size by 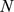 and the urban indicators by 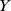. We thus want to check if 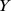 is a power law function of 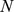, that is, 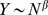, where 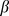 is the power law exponent. Figure 1 shows a scatter plot of 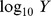 versus 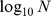 for all urban indicators, starting with the number of homicides and passing through all the ten urban metrics. We note that, despite the existence of considerable noise in some relationships, the scaling laws with the population size are perceptible. In order to overcome the noise and uncover the main tendency in these relationships, we have binned the data in 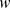 windows equally spaced in 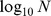 and evaluated the average values of the points within each window. The square symbols shown in Fig. 1 represent these average values and the dashed lines are linear fits. Note that linear functions describe quite well all the average relations, that is, the equation
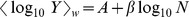
(1)holds for all the urban indicators. Here, 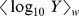 is the average value of 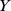 within each one of the 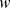 windows, 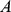 is a constant and 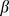 is the power law exponent (shown in Fig. 1). We have thus confirmed that there are scaling laws between the average values of the urban indicators 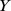 and the population 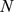. It is worth to remark that these average relationships are very robust when varying the number of windows 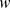 (see Fig. S1).

**Figure 2:**
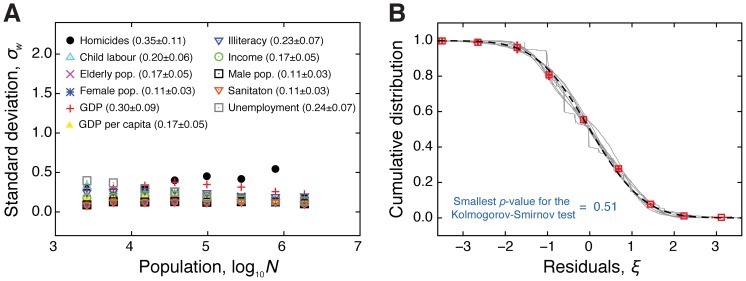
Fluctuations around the scaling laws. (A) Standard deviation 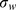 of the fluctuations around the scaling laws (in base-10 logarithmic scale) in each one of the 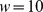 equally spaced windows. We note that the standard deviation is almost a constant function of the population for all urban indicators. The average value of 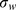 over the population windows are shown in the plot legends. (B) Cumulative distributions of the normalized fluctuations 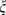 around the scaling laws. In this plot, each gray line is a distribution for a given indicator, the squares are the average values of these cumulative distributions and the error bars are 95% confidence intervals obtained via bootstrapping [44]. We note that the Gaussian distribution (dashed line) describes quite well these distributions. In particular, the smallest 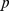-value of the Kolmogorov-Smirnov tests is 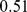, showing that we cannot reject the normality of the fluctuations.

Another striking feature of Fig. 1 is the fluctuation around the power law tendency. We have observed that the standard deviation
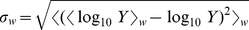
(2)within each window practically does not change with the population size 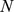 for all urban indicators (Fig. 2A). We have also verified that the normalized residuals around the power law,
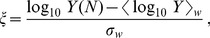
(3)are normally distributed with zero mean and unitary standard deviation (Fig. 2B). In particular, the Kolmogorov-Smirnov test [45] cannot reject the normality of 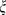 for all the urban indicators (the 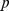-values are all larger than 0.51).

Our previous analysis thus enable an elegant formulation to the average scaling laws and also to the noise around these tendencies. Mathematically, we can write
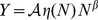
(4)


or, equivalently
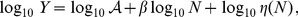
(5)where 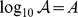 and 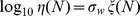. Notice that, since 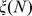 is normally distributed, 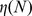 should be distributed according to a log-normal distribution. In addition to describe the average scaling laws, Eq. (4) represents a stochastic-like process where the urban indicator 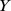 follows a power law relation with the population 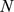 driven by a multiplicative noise log-normally distributed.

### Regression model: homicides versus urban metrics

As we have mentioned in the introduction, a considerable part of the literature about criminality tries to correlate crime indicators to other urban metrics. Usually, these relationships are obtained from linear regression models, despite the explicit nonlinearities present in these variables such as the previous scaling laws. In this context, it is not uncommon to observe linear regression-based analysis leading to controversial conclusions [34]. A simple alternative that may overcome these nonlinearities is to employ the logarithmic of the variables, that is,
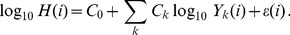
(6)


Here, 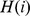 is the number of homicides in the city 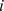, 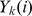 is the 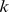-th (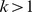) urban indicator of the city 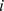, 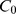 is the intercept coefficient, 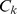 (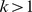) is the linear coefficient that quantifies the explicative effect of 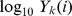, and 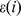 is the noise term accounting for the effect of unmeasurable factors.

We have applied the previous model to our data by using ordinary least-squares fit with a correction to heteroskedasticity [46] and the results are summarized in Table 1. We first note that, except for sanitation and unemployment, all the urban indicators have explanatory potential for describing the number of homicides. Also, the value of the adjusted 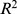 points out that the model account for about 62% of the observed variance in number of homicides. When analyzing the individual effects of the urban indicators, we note that child labour, elderly population, female population, GDP per capita, and male population are negatively correlated with the number of homicides (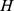 decreases with the increasing of these indicators). On the other hand, GDP, illiteracy, and income are positively correlated with the number of homicides (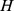 increases with the increasing of these indicators). Despite the lack of a more adequate comparison with our data, our regression results agree but also disagree with some empirical findings of the criminology literature. For instance, we have found that there is no statistically significant correlation between unemployment and homicides, while a positive and statistically significant correlation between illiteracy and homicides was observed. However, these indicators are among those leading to controversial conclusions, as pointed out by Gordon [34].

**Table 1:** Regression model coefficients.

*k*	Indicator *Y_k_*	Coefficient *C_k_*	Standard Error	*t*	p>|*t*|
		95% Confidence Interval			
Gray 0	Intercept	322.932	84.653	3.81	0.000
Gray		[156.944, 488.920]			
1	Child labour	−0.146	0.035	−4.11	0.000
		[−0.216, −0.076]			
Gray 2	Elderly population	−0.647	0.066	−9.81	0.000
Gray		[−0.777, −0.518]			
3	Female population	−56.644	15.488	−3.66	0.000
		[−87.015, −26.274]			
Gray 4	GDP	121.127	31.375	3.86	0.000
Gray		[59.605, 182.648]			
5	GDP per capita	−120.987	31.375	−3.86	0.000
		[−182.509, −59.465]			
Gray 6	Illiteracy	0.213	0.051	4.11	0.000
Gray		[0.111, 0.314]			
7	Income	0.223	0.073	3.05	0.002
		[0.079, 0.367]			
Gray 8	Male population	−62.459	16.068	−3.89	0.000
Gray		[−93.967, −30.952]			
9	Sanitation	−0.665	0.929	−0.72	0.474
		[−2.487, 1.156]			
Gray 10	Unemployment	−0.026	0.028	−0.94	0.347
Gray		[−0.082, 0.028]			
Adjusted *R^2^* = 0.62					

Values of the linear coefficients 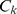 obtained via ordinary least-squares fits with a correction to heteroskedasticity. Here, 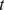 is the value of the 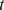-statistic and 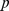 is the two-tail 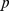-value for testing the hypothesis that the coefficient 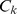 is different from zero.

Naturally, our regression model is quite simple and several improvements are possible. For instance, some of these metrics may display correlations and, consequently, one metric may affect the predicability of another, a phenomenon known as mediation [47]. A possible manner for reducing this effect is by combining some of the metrics and running different regression models. Another possibility is to employ principal component analysis (PCA) for reducing redundancy among the urban metrics. Nevertheless, other problems such as bias in the selection of urban metrics and difficulties in drawing qualitative conclusions in terms of the PCA axis are still present. Here, instead of discussing the possible controversies that Table 1 may exhibit as well as possible manner of improving our regression results, we will compare this simple regression analysis with our new approach based on the deviations of the scaling laws.

### A relative metric: distance to the scaling laws

In addition to overcome the nonlinearities by employing the logarithmic of the urban indicators, we may also account for the scaling behavior between the urban indicators, homicides and the population size (Fig. 1) aiming a fairer comparison between cities with different population sizes. We thus have proposed to evaluate the differences between the actual value of the urban indicators and the expected by the adjusted power law, that is,
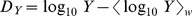


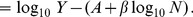
(7)


Note that 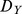 identifies whether a urban indicator for the given city is above (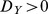) or below (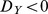) the average scaling law as well as how far it is. We have also evaluated this distance for the number of homicides, that is, 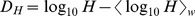 (note that we are committing an abuse of terminology when denoting 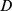 as a distance). This is the same idea recently proposed by Podobnik *et al.* [41] for quantifying the competitiveness among countries.

**Figure 3:**
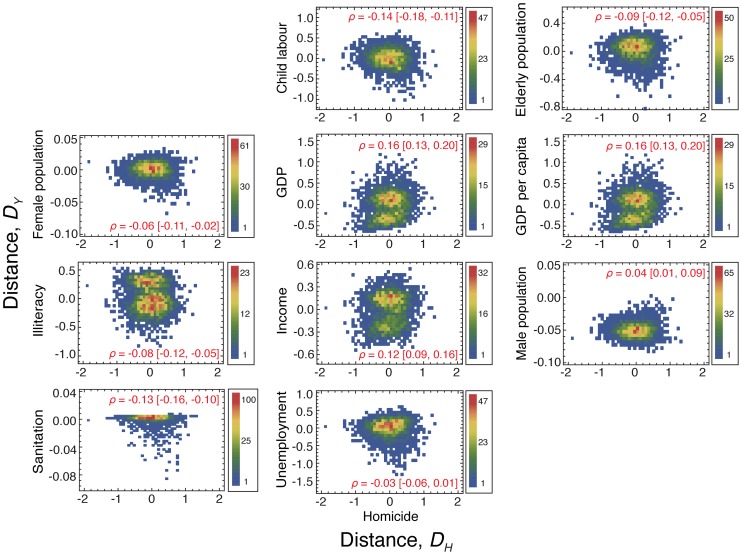
Distance to the scaling laws evaluated for the urban indicators versus the distance evaluated for the number of homicides. Scatter plot of the distances to the scaling laws evaluated for the urban indicators (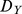) versus the distance evaluated for the number of homicides (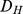). The color code represents the density of points, going from blue (low density) to red (hight density). We show in each plot the value and the 95% confidence intervals for the Pearson correlation coefficient 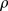. We note that 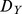 evaluated for GDP, GDP per capita, income, and male population are positively correlated with 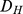, while 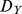 related to child labour, elderly population, female population, illiteracy, sanitation, and unemployment are negatively correlated with 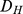. We further observe the bimodal distributions of the relationships for GDP, GDP per capita, illiteracy, and income.

We have thus studied the relations between the distance evaluated from the homicide indicator (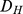) and the other urban metrics (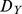). Figure 3 shows a scatter plot of 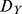 versus 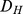, where we note that all of the urban metrics distances (except unemployment) have statistically significant correlations with the homicide distance (see the values of Pearson correlation 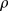 in these plots). We have also observed that the sign of the correlation coefficient 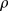 agrees with value of the linear coefficient 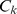 for the indicators child labour, elderly population, female population, GDP, income, sanitation, and unemployment. However, for the indicators GDP per capita, illiteracy and male population, the sign of 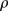 is opposite to the signal of 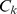. This result means, for instance, that while the regression analysis suggests that the increase in the male population is followed by a decrease in the number of homicides, the results when considering the relative distances point out that the more the male population is above the power law tendency, the more the number of homicides is above the power law tendency. Similar controversial conclusions are obtained for the indicators GDP per capita and illiteracy.

In addition to the value of the Pearson correlation 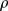, the scatter plots in Fig. 3 reveals other intriguing patterns. We note that the relation between the homicide distance and the indicators GDP, GDP per capita, illiteracy, and income are characterized by two peaks in the density of points, while for all the other indicators the density of points displays only one peak. We also note that both peaks of these bimodal distributions are located around 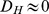. This result indicates that, despite the positive values of 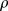, there is a considerable number of cities that displays distance values for 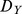 above and below the power law tendency with approximately the same value for the distance 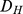, suggesting that such indicators may not be as good as the other ones for describing the number of homicides.

**Figure 4:**
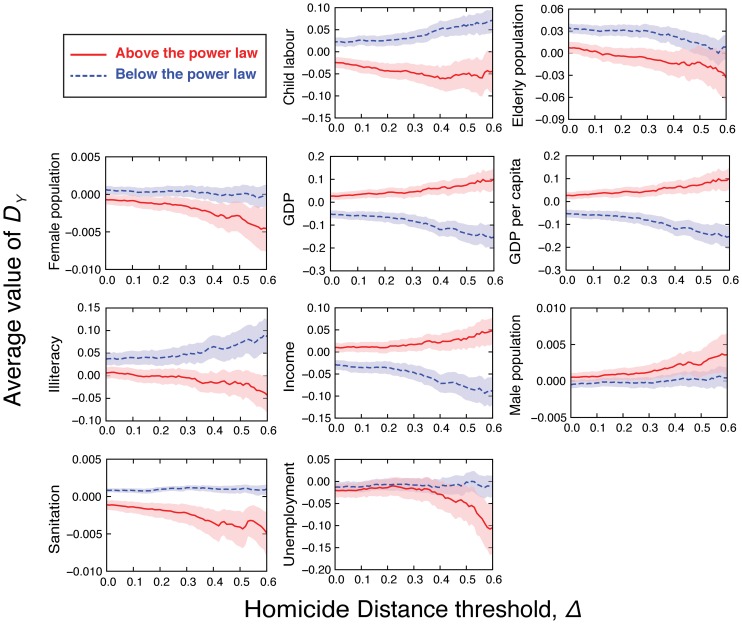
Average values of the distances to the scaling laws versus the homicide distance threshold. The average values of distances evaluated for each urban indicator in function of the homicide distance threshold 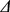, after grouping the cities that are above (red continuous lines) and below (blue dashed lines) the scaling laws with the population size. The shaded areas are 95% confidence intervals for these average values obtained via bootstrapping [44].

Another manner of extracting meaningful information from Fig. 3 is by evaluating average values. In order to do so, we have grouped the cities in two sets: those having 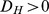 (homicides above the power law) and those with 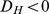 (homicides below the power law). We next evaluate the average value of 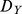 for each group and considering the cities with absolute value of 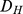 larger than a threshold 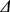. Figure 4 shows these average values as a function of the threshold 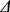. We have observed that for the indicators child labour, illiteracy and sanitation, the average values of 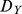 are significantly different between the two groups of cities and also that the average of 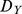 increases as 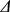 increases for the cities with 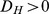 and decreases for those ones with 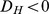. The opposite occurs for the indicators GDP, GDP per capita and income, that is, the average of 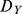 decreases as 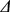 increases for the cities with 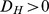 and increases for those ones with 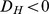. Intriguingly, for the indicator elderly population we observe that cities with 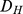 below the power law present an average value of 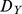 larger than those with 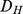 above the power law; however, this difference is only statistically significant for 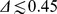. This result suggests that, for cities having a much larger or much smaller number of homicides than the expected by the power law tendency, the elderly population may have no explanatory potential. Similarly, for the unemployment indicator, no difference is observed between the average values of 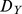 above and below the power law until 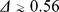. For slightly smaller value of 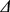, the average value of 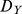 (for unemployment) for cities above the power law starts to systematically decrease and for 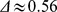 a statistically significant difference is observed. This result thus provides us a clue for a better understanding of the explicative potential of the unemployment indicator, by pointing out that (in our data) its effect is only manifested when 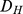 is much above of the value expected by the scaling law.

Figure 4 also provides clues of a gender effect in the number of homicides. For female population, we note that cities with number of homicides above the power law (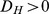) are characterized by an average value of 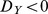 that decreases as the value of 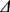 increases. We also observe that the confidence intervals for the average values of 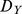 above and below the power law barely overlap each other. These results thus point out that in cities where the number of homicides is above the expected value, the female population is systematically smaller than the value expected by the scaling law. For male population, despite the overlapping in the confidence intervals for the average of 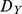, we observe an opposite behavior, that is, cities with number of homicides above the power law are also characterized by a male population above the power law.

## Summary and Conclusions

We have extensively characterized some relationships between crime and urban metrics. We have initially shown that urban indicators obey well defined average scaling laws with the population size and also that the fluctuations around these tendencies are log-normally distributed. Using these results, we have shown that the scaling laws can be represented by a multiplicative stochastic-like equation (Eq. 4) driven by a log-normal noise. Next, we have addressed the problem of applying regression analysis for explaining the number of homicides 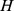 in terms of urban indicators 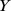. Because of the intrinsic nonlinearities, we have argued that it is better to employ the logarithms of these variables when performing linear regression analysis (Eq. 4 and Table 1). Furthermore, we have also discussed that accounting for the scaling phenomenon is also important for a fairer comparison among cities with different population sizes. We have thus proposed to evaluate the distances between the actual number of homicides 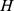 (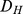) as well as the value of the urban indicator 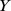 (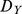) and the one expected by the average scaling laws. By investigating the Pearson correlations (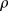) of the relationships between 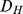 and 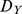, we have found that the value of 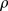 have the same signal of the linear coefficient 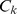 for the indicators child labour, elderly population, female population, GDP, income, sanitation, and unemployment. On the other hand, for GDP per capita, illiteracy and male population the signal of 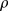 and 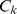 are opposite. In addition to the values of 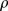, we have analyzed the average values of 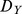 after grouping the cities in two sets: those with number of homicides above the power law (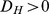) and those below the power law (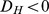). This analysis has unveiled intriguing patterns that were not carried out by the linear regression. In particular, our results for Brazilian cities pointed out that 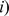 the elderly population may have no explanatory potential when the number of homicides is much above or much below of the expected values by the scaling law, 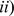 that the effect of unemployment in the number of homicides is only observed for cities with 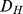 considerably larger than the expected by the power law, and 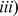 that there are gender differences in the number of homicides, where cities with female population below the expected value are characterized by a number of homicides above the power law and that cities with number of homicides above the power law are also characterized by a male population above the power law. We further believe that the present approach can be applied to other datasets in order to produce more robust relationships between crime indicators and urban metrics.

## Supporting Information

Figure S1
**Robustness of the power law exponent versus the number of windows employed in the average relationships.** The value of power law exponent 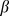 versus the number of windows 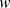 employed to evaluate the average relationships between 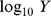 and 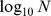. The error bars are 95% confidence intervals for the value of 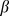 and the horizontal red lines are the average values of 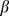 over 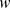. We note the almost constant behavior of 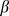 in function of 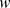.(TIF)

Table S1 **Data used in our analysis.** This comma separated file (CSV) has 13 columns where the first one contains the names of the cities and the other columns contains the urban metrics used here. The first line is a header indicating the urban **metrics.**
(CSV)

Text S1 **Details about the urban indicators used here.**
(PDF)
