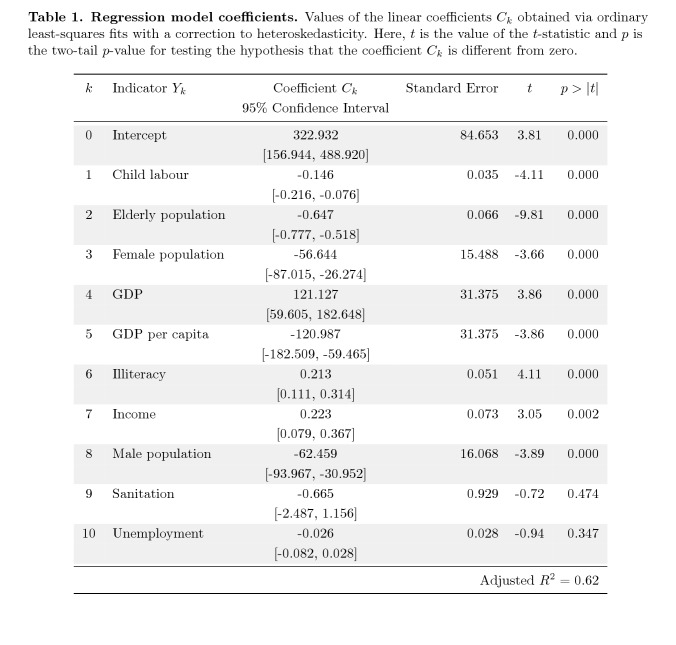# Correction: Distance to the Scaling Law: A Useful Approach for Unveiling Relationships between Crime and Urban Metrics

**DOI:** 10.1371/annotation/7471cfa3-9b0f-4cc8-8bc8-fd9fb3ce5844

**Published:** 2013-12-10

**Authors:** Luiz G. A. Alves, Haroldo V. Ribeiro, Ervin K. Lenzi, Renio S. Mendes

The word gray was erroneously added in the k column of Table 1. Please see the corrected Table 1 here: 

**Figure:**