# Supplementary figures and images for: Distance to the Scaling Law: A Useful Approach for Unveiling Relationships between Crime and Urban Metrics
Source: PLoS One. 2013 Aug 5;8(8):e69580. doi: 10.1371/journal.pone.0069580 (PMC3734155; doi:10.1371/journal.pone.0069580)

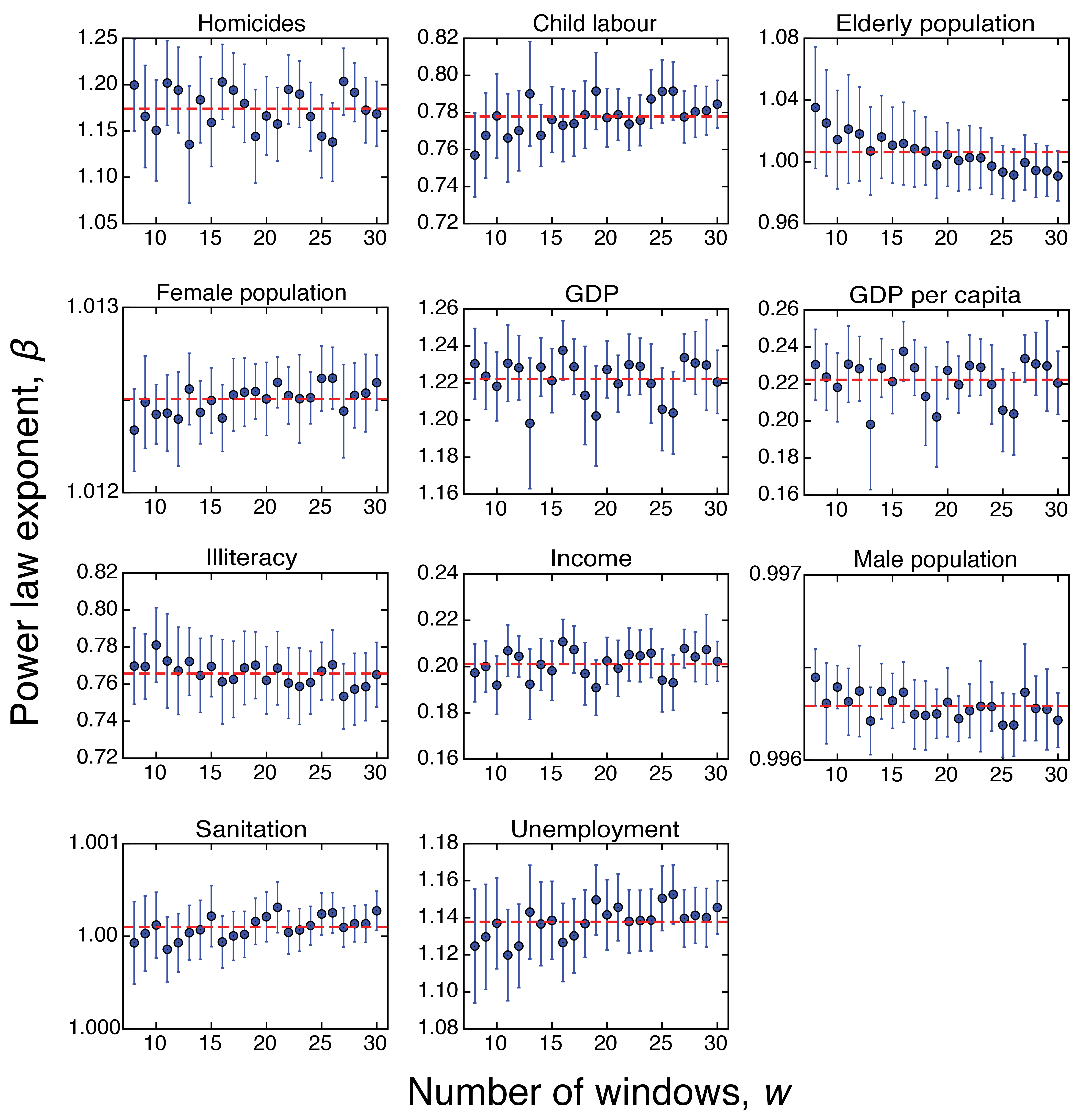

Supplement: Figure S1 — Robustness of the power law exponent versus the number of windows employed in the average relationships. The value of power law exponent versus the number of windows employed to evaluate the average relationships between and . The error bars are 95% confidence intervals for the value of and the horizontal red lines are the average values of over . We note the almost constant behavior of in function of . (TIF) [file pone.0069580.s001.tif]
