# Supplementary material for: Distance to the Scaling Law: A Useful Approach for Unveiling Relationships between Crime and Urban Metrics
Source: PLoS One. 2013 Aug 5;8(8):e69580. doi: 10.1371/journal.pone.0069580 (PMC3734155; doi:10.1371/journal.pone.0069580)
Supplement: Text S1 — Details about the urban indicators used here. (PDF) [file pone.0069580.s003.pdf]

Supporting Information: Distance to the scaling law: a useful approach for unveiling relationships between crime and urban metrics

Luiz G. A. Alves, Haroldo V. Ribeiro*, Ervin K. Lenzi, Renio S. Mendes
Departamento de Física and National Institute of Science and Technology for Complex Systems, Universidade Estadual de Maringá, Maringá, Brazil
∗ E-mail: hvr@dfi.uem.br

Supplementary Information

Definitions, data sources and additional comments

We have obtained data of all Brazilian cities in the year of 2000 made free available by the Brazil's public healthcare system — DATASUS [1]. Below, we describe the indicators and give some details about the data.

Homicide: injuries inflicted by another person with intent to injure or kill, by any means [2]. This indicator gives the number of deaths caused by assaults. We selected the death in the DATASUS website whose cause is included in the codes X85-Y09 from the International Classification of Diseases (ICD-10) [2].

Population: this indicator is derived from the population census of 2000 conducted by IBGE (Instituto Brasileiro de Geografia e Estatística) [3] and it reports the total number of inhabitants of each city. This database also contains information about age group and gender.

Illiteracy: it gives the number of inhabitants of the total population in a given geographic area, in the current year, aged 15 or older, who can not read and write at least a single ticket in the language they know.

Income: this indicator gives the average household incomes per capita of residents in a given geographic area, in the current year. It was considered as per capita household income the sum of the monthly income of the household, in reals divided by the number of its residents.

Unemployment: it gives the number of economically active population aged 16 or older who is without work during the reference week, in a given geographic area, in the current year. It is defined as the Economically Active Population (EAP) the number of persons aged 10 or older who are working or looking for work. For this indicator, it was considered only the population aged 16 or older.

Child labour: the proportion of the population 10 to 15 years old who is working or looking for work during the reference week, in a given geographic area, in the current year.

GDP per capita: Gross Domestic Product (GDP) per capita indicator is the value of the municipal GDP per capita, being calculated as the municipal GDP of the year divided by the municipal population in the same year. The values are presented in the currency real, not being applied deflator or no correction factor .

GDP: it gives the value of the municipal GDP. Values are given in thousands of the currency real, not being applied deflator or no correction factor .

Elderly population: the number of inhabitant of a given city aged 60 or older.

Sanitation: it gives the number of inhabitants that has access to toilets, garbage collection and water supply .

References

1. Brazil's Public healthcare System (SUS), Department of Data Processing (DATASUS). Available: http://www.datasus.gov.br/. Accessed 2013 Feb 1.

2. World Health Organization (WHO). Available: http://apps.who.int/classifications/icd10/. Accessed 2013 Feb 1.

3. Brazilian Institute of Geography and Statistics (IBGE). Available: http://www.ibge.gov.br/. Accessed 2013 Feb 1.
